# Supplementary material for: Tissue-Specific Vulnerability to Apoptosis in Machado-Joseph Disease
Source: Cells. 2023 May 17;12(10):1404. doi: 10.3390/cells12101404 (PMC10216795; doi:10.3390/cells12101404)
Supplement: Supplementary file 1 [file cells-12-01404-s001.zip › cells-2362530-supplementary.pdf]

Supplementary Table S1. Characterization of the MJD subjects (preclinical subjects and patients) and control individuals used in this study (n=124).

	Preclinical subjects	Patients	Controls
**Blood samples**			
n (Female; Male)	19 (12; 7)	37 (19; 18)	54ᵗ (30; 24)
Age[1], years	30.3 ± 7.4 [21; 44]	45.9 ± 11.6 [26; 65]	41.1 ± 12.7 [21; 67]
CAG_n allele 1[2]	20.0 ± 4.1 [14; 28]	20.7 ± 4.9 [14; 29]	19.6 ± 4.3 [14; 27]*
CAG_n allele 2[3]	68.1 ± 3.0 [62; 75]	71.2 ± 2.8 [64; 76]	24.0 ± 4.0 [14; 32]*
Years to onset[4], years	-10.5 ± 9.2 [-26; +5]	NA	NA
Age at onset, years	NA	35.2 ± 7.9 [22; 52]	NA
Disease duration, years	NA	10.7 ± 8.7 [1; 36]	NA
**Post-mortem brain samples**			
n (Female; Male)	NA	5 (4; 1)	9 (5; 4)
CAG_n allele 1[2]	NA	19.0 ± 4.1 [12; 22]	17.0 ± 5.3 [11; 25]
CAG_n allele 2[3]	NA	70.2 ± 3.0 [66; 73]	22.6 ± 5.6 [12; 30]
Age at onset, years	NA	45.0 ± 8.5 [39; 51]**	NA
Disease duration, years	NA	26.5 ± 9.2 [20; 33]**	NA
Age at death, years	NA	63.0 ± 16.0 [48; 84]	69.6 ± 12.2 [48; 83]
PMI[5], hours	NA	26.6 ± 17.2 [4; 48]	15.4 ± 8.0 [4; 24]

Quantitative variables are displayed as mean ± standard deviation [minimum; maximum]; [1]Age at first blood collection, [2]Number of CAG repeats in the normal allele of MJD subjects/number of CAG repeats in normal allele 1 of controls; [3]Number of CAG repeats in expanded allele of MJD subjects/number of CAG repeats in normal allele 2 of controls; [4]Years to onset: negative values indicate the numbers of years missing to the estimated onset and positive values indicate the number of years that have elapsed the estimated onset; [5]Post-mortem interval; ᵗAge (±3 years) and sex-matched paired controls for preclinical subjects and patients (two individuals were used as paired matched controls for both groups); *Information available for 47 controls; **Information available for two patients; NA, not applicable/not available

Supplementary Table S2. Demographic, genetic, and clinical data of the 18 MJD patients used in the follow-up study.

	Baseline	Visit 1	Visit 2
n (Female; Male)	18 (7; 11)	18 (7; 11)	11 (4; 7)
Age[1], years	48.9 ± 13.7 [26; 65]	53.9 ± 13.6 [32; 72]	52.6 ± 13.9 [34; 72]
Normal CAG allele	19.7 ± 5.1 [14; 29]	19.7 ± 5.1 [14; 29]	19.6 ± 5.9 [14; 29]
Expanded CAG allele	70.9 ± 3.2 [64; 76]	70.9 ± 3.2 [64; 76]	71.4 ± 3.9 [64; 76]
Age at onset, years	36.2 ± 7.9 [22; 50]	36.2 ± 7.9 [22; 50]	36.1 ± 9.0 [22; 50]
Disease duration, years	12.7 ± 9.5 [1; 36]	17.7 ± 9.4 [7; 40]	16.6 ± 6.7 [9; 27]

Quantitative variables are displayed as mean ± standard deviation [minimum; maximum]; [1]Age at first blood collection

Supplementary Table S3. Characterization of post-mortem human brain samples from MJD patients and controls individuals, and RNA integrity number of each brain samples used in this study.

Health condition	Sex	ID	Age at death (years)	PMI[1] (hours)	Cause of death	Age at onset (years)	CAG repeats	RNA integrity number		
							Allele 1 | Allele 2	DCN[2]	Pons	Frontal cortex
Controls	Female	1	48	5	Polycythemia vera, mesenteric thyrombosis and ischemic bowel resection	NA	12|19	3.7	3.2	3.1
		2	76	14	Cardiac failure	NA	21|25	2.9	5.3	3.4
		3	80	19	Congestive heart failure and atrial fibrillation	NA	12|18	5.2	6	NA
		4	83	NA	Renal cell carcinoma	NA	18|21	4.8	4.6	5.6
		5	83	21	Cardiac arrest, urinary tract infection and sepsis	NA	11|12	6.9	5.7	NA
	Male	6	59	12	Sudden cardiac arrest, ventricular fibrillation and post-shock electromechanical dissociation	NA	12|25	7.7	NA	6.5
		7	61	24	Cardiac failure, cardiogenic shock and post-shock electromechanical dissociation	NA	21|25	7.5	NA	6.9
		8	65	24	Acute respiratory distress syndrome and sepsis	NA	25|28	NA	NA	NA
		9	71	4	Cardiac failure	NA	21|30	7	7.4	8.3
MJD patients	Female	10	48	22	NA	NA	22|73	5.3	4.1	6.2
		11	59	4	NA	39	21|70	7.9	6.2	8.4
		12	75	39	NA	NA	19|69	NA	NA	4
		13	84	20	NA	51	21|66	6.5	NA	4.7
	Male	14	49	48	NA	NA	12|73	NA	3.5	3.9

[1]*Post-mortem* interval; [2]Dentate cerebellar nucleus; NA, not applicable/not available

Supplementary Table S4. Genotypes of the 9 and 18 month-old mice used in this study.

Age	Genotype	Gender	Mouse #	Mouse Tag	CAG repeats
9-month-old	wt[1]	Female	1	840.0.0	NA
			2	840.0.2	
			3	817.0.0	
			4	817.0.3	
			5	843.0.1	
			6[#]	840.0.4	
		Male	7	842.0.1	NA
			8	845.0.1	
			9	845.0.2	
			10	839.0.2	
			11*	846.0.2	
			12*	855.0.0	
	Q84[2]	Female	13	817.0.1	73/74/**76**/83
			14	817.0.2	**73**/76/79/88
			15	840.0.1	**72**/75/84
			16	817.0.4	68/**73**/80/83
			17	843.0.0	68/**73**/79/82
			18	843.0.3	**71**/75/78/85
		Male	19	813.0.0	68/**73**/77/82
			20	813.0.1	68/**73**/77/83
			21	842.0.2	**71**/75/81
			22	845.0.0	**72**/76/86
			23*	845.0.3	**72**/77/85
			24[#]	842.0.0	NA
18-month-old	wt[1]	Female	25	61.0.0	NA
			26	61.0.1	
			27	691.0.3	
			28	702.0.0	
			29	702.0.1	
	Q84[2]		30	61.0. 4	NA
			31	691.0.1	
			32*	702.0.2	
			33	710.0.3	

[1]wild type littermate mice; [2]hemizygous YACMJD84.2 transgenic mice; [#]Protein sample not available; *RNA sample not available; NA, not available; the main CAG allele is indicated in bold

Supplementary Table S5. Correlations between transcript levels of *BCL2*, *BAX* and *TP53*, as well as *BCL2*/*BAX* ratio and demographic, genetic and clinical data of MJD subjects (preclinical individuals and patients).

					Age at onset		DD[3]	
		**Age[1]**	**CAG-E[2]**	**Years to onset**	**CAG-E adj.**	**Age + CAG-E adj.**	**No adj.**	**Age[1] adj.**
**Preclinical subject**								
BCL2	Rho	0.269	0.292	-0.275	NA	NA	NA	NA
	Sig (2-tailed)	0.266	0.225	0.255				
BAX	Rho	-0.001	0.052	0.002	NA	NA	NA	NA
	Sig (2-tailed)	0.997	0.832	0.994				
TP53	Rho	-0.090	0.078	0.101	NA	NA	NA	NA
	Sig (2-tailed)	0.715	0.751	0.681				
BCL2/*BAX*	Rho	0.005	0.217	0.093	NA	NA	NA	NA
	Sig (2-tailed)	0.985	0.403	0.722				
**Patient**								
BCL2	Rho	-0.264	0.268	NA	0.011	NA	0.229	NA
	Sig (2-tailed)	0.114	0.109		0.951		0.173	
BAX	Rho	-0.350	0.273	NA	-0.522	-0.482	0.048	0.308
	Sig (2-tailed)	**0.034**	0.102		**0.001**	**0.003**	0.777	0.067
TP53	Rho	-0.360	0.295	NA	-0.189	-0.106	0.198	0.090
	Sig (2-tailed)	**0.029**	0.076		0.270	0.544	0.240	0.600
BCL2/*BAX*	Rho	0.243	-0.222	NA	0.403	0.393	0.014	0.279
	Sig (2-tailed)	0.153	0.194		**0.016**	**0.022**	0.933	0.105

[1]Age at first blood collection; [2]CAG-E: expanded CAG repeat;[3]Disease duration

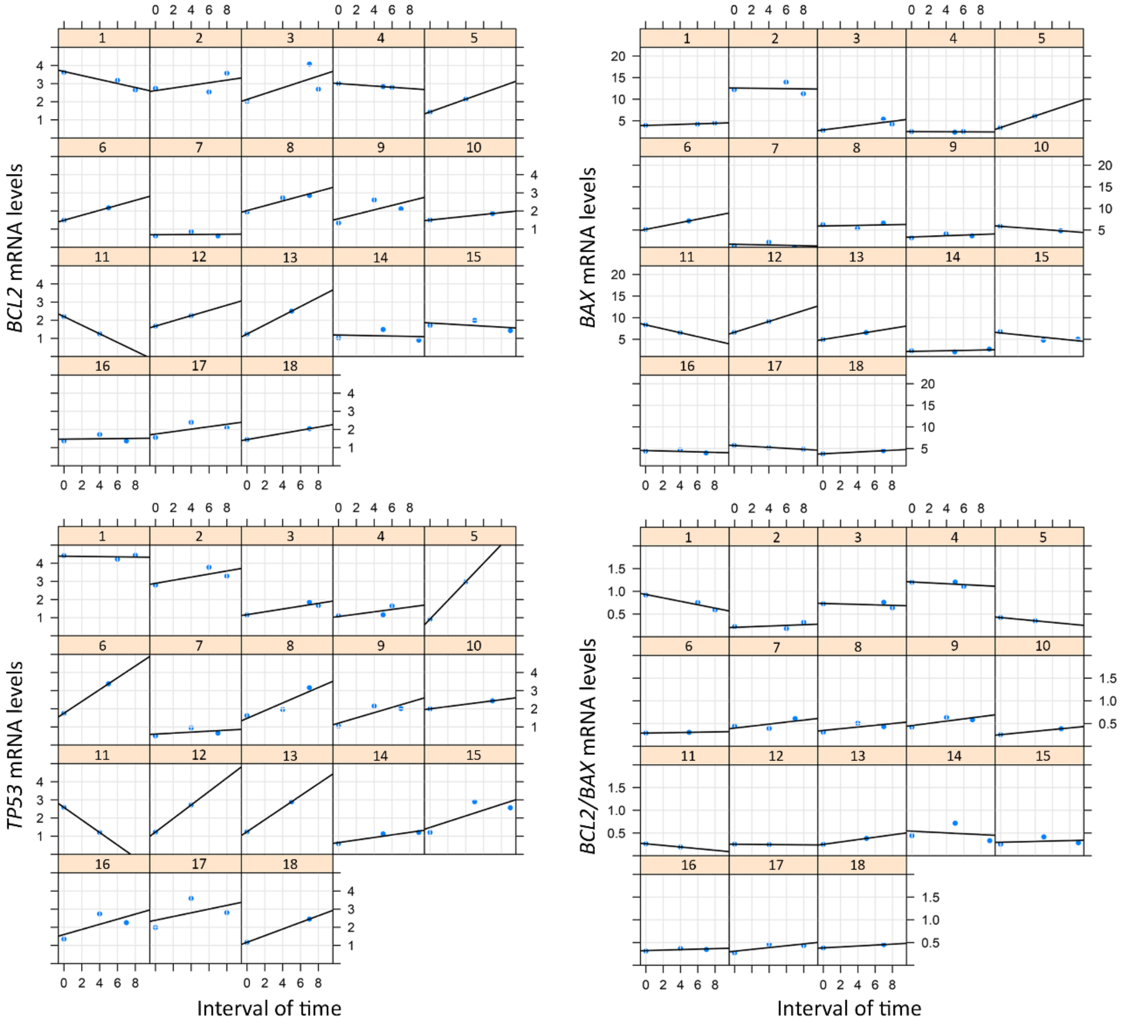

Supplementary Figure S1. *BCL2*, *BAX* and *TP53* transcriptional levels and *BCL2/BAX* ratio changes over time in the 18 patients analyzed in the follow-up study.

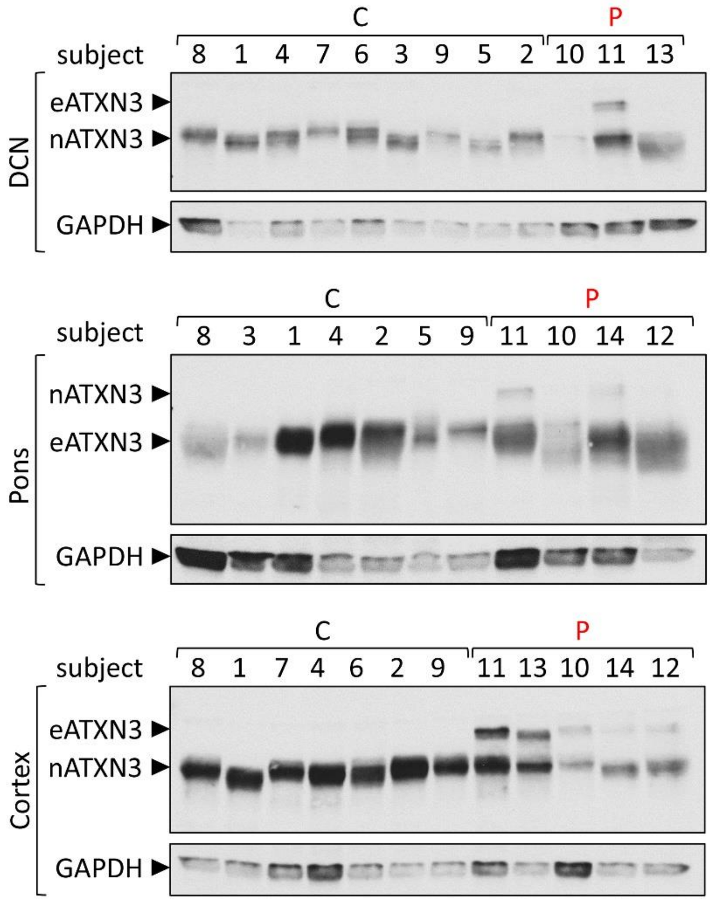

Supplementary Figure S2. Western blot using the anti-ATXN3 antibody (1H9) to detect the native human ATXN3 (nATXN3) and expanded human ATXN3 (eATXN3) in insoluble protein fraction of post-mortem human samples from dentate cerebellar nucleus (DCN), pons and frontal cortex (Cortex) of Machado-Joseph disease patients (P) and control subjects (C). GAPDH was used as a protein loading control.

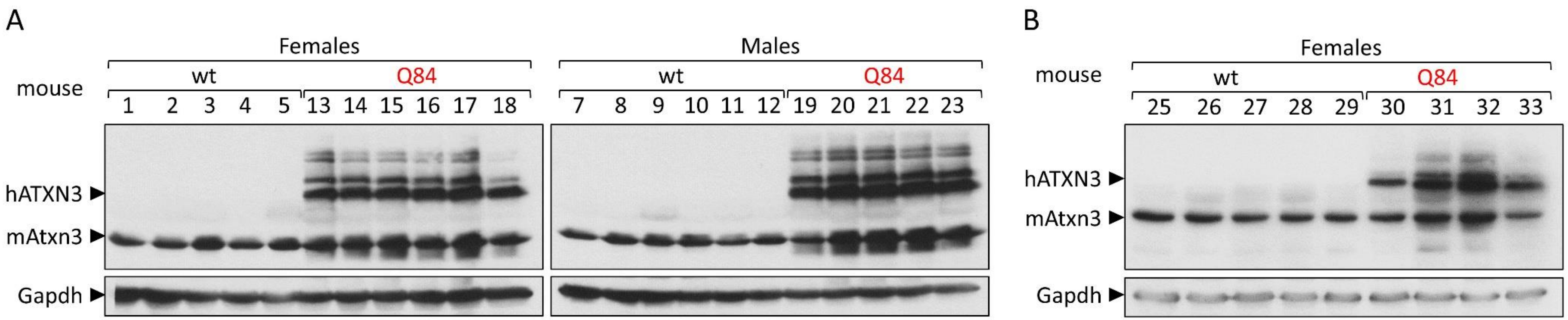

Supplementary Figure S3. Western blot using the anti-ATXN3 antibody (1H9) to detect the mutant human ATXN3 (hATXN3) and endogenous mouse ATXN3 (eATXN3) in soluble fraction protein from (A) cerebral cortex of 9 months-old and (B) 18 months-old hemizygous YACMJD84.2 (Q84) transgenic and wild-type (wt) littermate mice. GAPDH was used as a protein loading control.
